# Process-based measures in high-stakes testing: practical implications for construct validity within military aviation selection

**DOI:** 10.1186/s41235-025-00660-3

**Published:** 2025-08-20

**Authors:** Joseph T. Coyne, Laura Jamison, Kaylin Strong, Ciara Sibley, Cyrus Foroughi, Sarah Melick

**Affiliations:** 1 Informaiton Technology Division, Naval Research Laboratory, Washington, DC USA; 2 Strategic Analysis, Arlington, VA USA; 3 Operational Psychology Department, Naval Aerospace Medical Institute, Pensacola, FL USA

## Abstract

This paper looks at how process-based spatial ability and attention measures taken within a high-stakes battery used to select pilots in the US Navy compare to lab-based measures of the same constructs. Process-based measures typically function by having individuals perform either a novel task or perform a task with novel stimuli. However, applicants often spend time practicing the tasks prior to taking the battery. A group of 307 Naval Flight Students participated in the study, in which they took several spatial ability, attention and general processing measures. One of the spatial tasks used in the study was the same as the spatial task in the Navy’s pilot selection battery, which all of the participants had taken. All of the lab spatial ability measures including the one used in the selection battery were highly correlated and loaded onto the same spatial ability factor. However, the high-stakes spatial subtest was not correlated with any of the lab spatial measures including the same test administered in the lab. The lab spatial ability data was also correlated with training outcomes whereas the high-stakes process spatial and attention measures were not. The high-stakes attention measure was weakly correlated with some of the general processing measures. The pattern of results suggest that familiarity with the spatial and attention tasks in the high-stakes environment may be negating those tests ability to measure the constructs they were designed to measure, and also reducing their effectiveness to predict training performance.

**Statement of significance:** This paper addresses an increasingly difficult challenge the Navy is facing within aviation selection, in that applicants are highly motivated and have access to unofficial replicas of the Navy’s test battery. The challenge is specific to the process-based measures such as spatial ability and attention that rely on some degree of novelty to work. When applicants practice these types of tests they can practice to the test, memorize items, and learn strategies which impact the test’s ability to measure the cognitive construct it was designed to measure as well as reduces its ability to predict flight training outcomes. This is particularly problematic as the unofficial test preparation software can replicate a new test within days. While the data presented here are limited to spatial ability and attention within military pilot selection it applies to a much broader community of researchers. Anyone developing a high-stakes test with a large and motivated applicant pool may also see their process-based measures perform differently in a high-stakes environment than a low stakes laboratory one in which participants are naïve to the tasks they are taking. The extent to which practice can alter the effectiveness of high-stakes test performance is an important one. The results of the paper suggest that test developers should assume participants are practiced and assess the extent to which practice on process-based measure impacts the tasks ability to measure the construct of interest and predict performance.

## Introduction

Each year approximately 6000 applicants take the Aviation Selection Test Battery (ASTB) to determine whether they have the aptitude to become a pilot or flight officer in the US Navy, Marine Corps and Coast Guard. One of the unique challenges faced in this selection procedure is that selection decisions are not based upon flight experience, but rather an individual’s aptitude to learn to fly. Those applicants are competing to be one of about 1100 pilots who the Navy trains each year (Prasad-Rao et al., 2023). The majority of applicants who take the ASTB do not achieve qualifying scores. Given the consequences of the battery, the ASTB is a high-stakes test particularly for those individuals who have a strong desire to pursue an aviation career within the military.

Beyond the individual’s consequences for performing on the battery, the effectiveness of the test is critical to the Navy. The U.S. Government Accountability Office (GAO, 2018) estimated it cost the military between $3-$11 million and approximately 5 years to train a single fighter pilot. The combined attrition rate for all phases of flight training is between 15 and 20% for Naval Aviators (NAMI, 2024). The cost of attrition varies by platform (e.g., helicopter, tilt–rotor, and strike) and the stage of training (Prasad-Rao et al., 2023). By the time a pilot reaches primary flight training the cost is $250 k per individual. Those costs quickly escalate as training moves to more expensive airframes with increased fuel and maintenance costs. It is estimated that without the ASTB the rate of attrition would be double (Arnold, 2002). Therefore, ensuring the battery retains and improves its predictive validity is critical to the Navy.

Like most aptitude assessments the ASTB is a combination of fluid and crystalized intelligence, or process and content knowledge, respectively. The content measures include: math, reading, mechanical, aviation and nautical knowledge. The process measures are all part of the Performance Based Measures portion of the test which contains ability tests (e.g., attention, psycho-motor, and spatial ability). The performance tests were developed to better reflect the “dynamic environment inside the cockpit” which traditional pen and paper tests can miss (Walker et al., 2007). This paper focuses on the measurement of process related abilities, specifically spatial ability and attention as they are measured within the ASTB. These constructs are also used by the US Air Force in their pilot selection battery the Test of Basic Aviation Skills (TBAS). The overall batteries are similar and the test of spatial ability is the same. Beyond pilot selection, the US military in general is interested in measurement of these constructs for determining eligibility for enlisted jobs. Attention and spatial ability were two of the three new predictor constructs that the National Research Council recommended to the US Army for consideration in enlisted selection and classification (NRC, 2015). These constructs are seen to have predictive validity, do not require special equipment, and can add incremental validity to selection batteries which are largely comprised of measures of crystalized intelligence (NRC, 2015).

Ackerman (2022) discusses two related problems associated with process measures in intelligence testing, familiarity and learnability. Process related measures involve either a novel process, novel stimuli, or both. Ackerman argues that an individual’s level of familiarity with the stimuli can impact their performance on the test, and therefore process measures can be confounded when individuals vary in their familiarity. The second problem he discusses is learnability, the degree to which performance will improve with repeated exposure to the task. Ackerman points to the reduction in correlations between initial exposure and final exposure as the number of exposures increases over time.

The issues raised by Ackerman (2022) are amplified in high-stakes environments such as the ASTB. There are a number of unofficial resources available to ASTB applicants which recreate subtests within the battery for applicants to practice. This paper includes the first large scale assessment of a new spatial ability measure, the Terrain Orientation Task, which we will refer to as the terrain task throughout this paper. The terrain task was added to the ASTB in September 2023, and within three weeks a practice version of the task was available in an unofficial test prep app. The stimuli in the practice version are different from the ASTB; however, the unofficial version allows applicants to practice the process. An assumption should be made that motivated and informed test takers will have direct exposure to portions of the ASTB prior to having ever taken the exam. A challenge for administrators of high-stakes tests is that there is currently no way of measuring an individual’s familiarity with the test. This familiarity potentially reduces the battery’s effectiveness by adding unknown variance. What is more problematic is that there are strategies to some process tests within the ASTB which may negate or reduce its ability to measure the construct it was designed to measure.

A review on spatial ability or even the use of spatial ability within aviation selection are outside the scope of this paper. Lohman’s (1993, p. 1) definition of spatial ability “the ability to generate, retain, retrieve, and transform well-structured visual images whose properties include location, size, distance, direction, separation, connection, shape, pattern, and movement” is a widely accepted one. Spatial ability is a multifaceted-construct, one in which there is some disagreement not only on what the components are but also which are the most important in careers such as aviation (e.g., Baron & Rose, 2013). Measures of spatial ability have been included in military aviation selection since World War I (Damos, 2011). This paper focuses on how spatial ability is currently measured, and how high-stakes testing influences the effectiveness of those measures.

The Direction Orientation Task (Fig. 3) is a spatial ability test designed by the US Air Force (Carretta, 2005), and is currently still a part of their pilot selection battery. Baron and Rose (2013) found that the Direction Orientation Task significantly predicted pilot training outcomes in the Air Force. The test was adopted by the Navy, and included in the ASTB in 2013. The Direction Orientation Task was initially predictive of training outcomes for the Navy, however, more recent analysis demonstrated that the test had lost its incremental validity over time (Coyne et al., 2022). Prior to its inclusion in the ASTB, Momen (2009) looked at retest performance of the Direction Orientation Task and found a significant improvement in performance across the two attempts on consecutive days. However, the assumption was made that practice effects would decrease over time as applicants would not be allowed immediately retake the ASTB. The Navy and Air Force both limit re-testing such that a minimum number of weeks must pass between test attempts. This testing policy is meant to limit the practice effects from exposure to the official test. However, this policy does not account for the fact that applicants have ways to unofficially practice between test administrations.

Each Direction Orientation Task trial has two images, one which shows the heading of an aerial vehicle (12 possible orientations based upon 30 degree heading increments) and the other image corresponds to a camera from that vehicle depicting four parking lots surrounding a building. The four parking lots are located at the four cardinal directions (North, East, South and West). The trial asks the individual to select one of the four parking lots (e.g., West). The position of that parking lot within the image depends on the heading of the aircraft. When the vehicles heading is one of the cardinal directions the parking lot on the top of the second image is the same as the heading. One of the limitations of the Direction Orientation Task is there are only 48 test items, individuals see every item, and the items are easy to replicate for practice purposes. Attempts to develop a more difficult version of the task, revealed additional concerns regarding non-spatial strategies (Keiser et al., 2019, Coyne et al., 2020). When surveyed on strategies used after completing the newer more difficult versions of the Direction Orientation Task, it was revealed that there was a negative correlation with the degree to which participants reported using a spatial strategy and performance, and a strong positive correlation between the use of math strategies and performance. These modified Direction Orientation Tasks were never included in the ASTB; however, these findings are problematic as they demonstrate a challenge with spatial ability tests which are amplified in a high-stakes testing. The concern is that effective non-spatial strategies are shared in online forums, and then adopted by a large subset of the population. A simple mathematical solution to the task is to compute the difference between the heading and the target parking lot, in Fig. 3 the heading 120 degrees is 60 degrees away from the target parking lot (south/180) and the response parking lot’s position is at 60 degrees. Another non-spatial solution is to draw a compass and then rotate it to match the heading of the vehicle. The Direction Orientation Task likely added incremental validity initially as it measured spatial ability, however as applicants became familiar with the items and aware of non-spatial strategies the test lost its effectiveness.

**Fig. 1:**
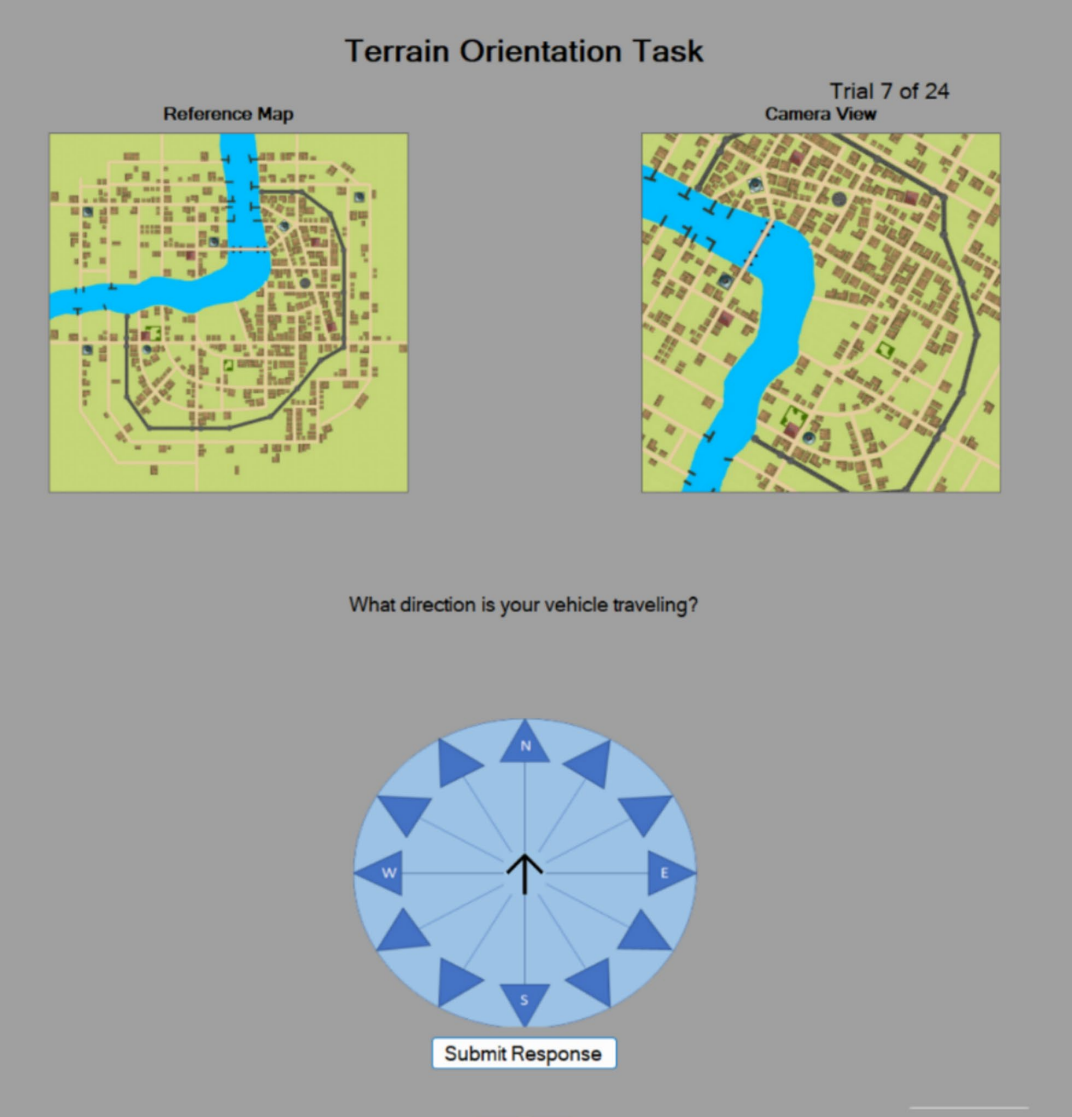
Sample trial for the Terrain Orientation Task (correct answer is 30 degrees)

The Terrain Orientation Task (Fig. 1) was developed as a potential replacement for the Direction Orientation Task. The Terrain Orientation Task addresses a number of limitations of the Direction Orientation Task. The Terrain task has an unlimited number of items a single map can serve as 12 items and there is an infinite pool of potential maps. More importantly the task does not have a mathematical solution similar to the Direction Orientation Task. The task does not provide any numerical values (no heading and no target position). Rather the problem most be solved by comparing differences in orientation between the two images. The Terrain Orientation Task is a terrain association task which provides a reference map and a “camera view” of the same terrain in a different orientation. An initial evaluation of the task found that it was significantly correlated with initial training performance in a group of Naval Flight Students (Coyne et al., 2024). The Terrain Task was designed to capture the same spatial ability variance as the Direction Orientation Task, however in the initial evaluation performance on Terrain Orientation Task (lab) and scores on the Direction Orientation Task taken within the ASTB (high-stakes) were not correlated. The present study addresses whether these two process tests measure the same construct.

The first goal of this work was to evaluate whether a new measure of spatial ability, the Terrain Orientation Task, could predict training performance for Naval Flight Students. Previous research in a small sample had demonstrated that Terrain Task could predict grades in ground school. However, that sample was small (*N* = 114) and the study did not compare the Terrain Orientation Task to other spatial ability measures. The current study is comprised of a larger sample of flight students and includes several measures of spatial ability. All of the flight students would have taken the ASTB, and would thus have familiarity with the Direction Orientation Task. This study took place prior to the Terrain Orientation Task being included in the ASTB so this was the students first exposure to the new test. It is hypothesized that the Terrain Task, and other spatial ability measures would predict training outcomes for the flight students. However, given the participants familiarity with Direction Orientation Task, it was hypothesized that the Direction Orientation Task in the study would not predict training outcomes or significantly correlate with the other spatial ability measures.

Spatial ability is only one of several process-based constructs measured in the ASTB.

A second objective of the study was to assess whether new measures of attention control could predict training outcomes. The ASTB includes a Dichotic Listening Task which is intended to measure selective attention. The task has individuals attend to information in one ear (e.g., responding to numbers and ignoring letters) while ignoring information simultaneously presented in the other year. The ear to which individuals must attend switches throughout the task. This type of task was first developed by Gopher and Kahneman (1971) and was used in selection of Israeli pilots. Similar to the DOT, the authors are aware of strategies on the Dichotic Listening Task which could eliminate or reduce the attentional demands of the task.

Attention control is “a domain general cognitive ability that plays a role in most controlled mental operations” (Burgoyne et al., 2023). Research in individual differences in attention control has received increased interest since a pair of recent papers identified measurement issues related to response time difference scores which limited the reliability of traditional measures (Hedge et al., 2018, Draheim et al., 2019). These newer measures focus on accuracy and have been shown to reliably measure the construct (Draheim et al., 2021). Burgoyne et al. (2025) described attention control as a “bottleneck that constrains performance across cognitive domains.” Failures to control one’s attention can impede an individual’s ability to learn new information, and can hinder performance within complex tasks such as flying. 

Recently, several of these measures of attention control have been shown to predict training performance in Navy Air Traffic Controllers (Burgoyne et al., 2025; Coyne et al., 2024). Initial research on attention control with Navy flight students collected prior to the COVID-19 pandemic found that several longer measures of attention control predicted flight training outcomes (Burgoyne et al., 2025). However, these tests which took over 30 min of time and were considered too long to be included in the Navy’s selection battery. Researchers at Georgia Institute of Technology focused on the development of three shorter measures of attention control, specifically double conflict versions of the Stroop, Flanker, and Simon tasks, in which conflict could be present in both the stimulus and the response (Burgoyne et al., 2023). An evaluation of these three new tests revealed that the Stroop and Flanker tests were predictive of flight training outcomes in a group of Navy flight students; however, the Simon test was not (Coyne et al., 2024). This study continues to examine the Stroop and Flanker tasks for potential use within the ASTB. The second hypothesis is that these two new measures of attention control will correlate with other domain general cognitive processing (e.g., working memory and fluid intelligence tasks) and predict training outcomes. It is expected that the familiarity with the high-stakes Dichotic Listening Task will reduce its capability to capture the same domain general variance that the new attention tasks capture.

## Method

### Sample

Data were collected from 307 NFS between 2021 and 2022. The mean age was 23.5 (SD = 2.4) and 58 of the students were female. The students were all Naval Officers, the majority of whom were training to be Naval Aviators, the remaining students were training to be Naval Flight Officers. All of the students had taken the ASTB and are part of a down selected sample. The students all participated in the study prior to beginning their flight training. All of the students were waiting to begin the Naval Introductory Flight Evaluation (NIFE) which is the Navy’s introductory training and has a ground school component as well as smaller flight component. Aviators and Flight Officers take the introductory training together following the same syllabus. All participants consented to the research study, and consented to have their lab data linked to both their ASTB data and training outcome data. Participants were all told their performance in the lab would not be used in any decisions with respect to their own careers, but would be used to inform the design of future versions of the ASTB. The study was approved by the primary author’s institutional review board.

It is worth noting that this sample was representative of the population of interest, highly motivated Naval Officers training to become Naval Aviators or Naval Flight Officers. In some studies on military personnel, corrections for range restriction are applied because the full population is not adequately represented and reported correlations may be underestimated since only selected personnel are evaluated (Carretta & Ree, 2022). However, for the current study, we are specifically interested in the latent structure of these measures in a highly motivated population where participants are likely to have practiced and prepared for the test. Our aim is to estimate the predictive capacity of these measures only for individuals who are already flight students. Therefore, we do not apply any range restriction corrections using other populations (e.g., all ASTB test takers) since the raw sample correlations represent an accurate cross section of our population of interest.

### Procedure

The test protocol was approximately 90 min. Participants came to the lab in groups of 10 and completed the tasks in the same order. The tasks were programmed in C#, XOJO, and E-Prime. A wrapper was programmed in C# that forced the participants to start each task in order allowing them to move through the tasks at their own pace. Participants completed a brief demographic survey followed by a series of spatial ability tasks, the terrain task, Spatial Apperception Task, the Direction Orientation Task, Cube Comparison and Paper Folding. After the spatial tasks participants completed the Mental Counters Task, Stroop and Flanker Double conflict tasks, and the Raven’s Advanced Progressive Matrices.

### Terrain orientation task/terrain task

The Terrain Orientation Task (Fig. 1) is designed to measure terrain association that is the ability to identify landmarks and use their position relative to other objects to determine orientation. Individuals are told that the reference map on the left is always oriented with North at the top. They are told that the camera map is an aerial view of the same area but from a vehicle traveling on a different heading. The goal is to determine the vehicles direction based upon changes in landmark/feature positions across the two images. For example in the sample trial in Fig. 1 there is a single East/West spanning bridge that crosses the river. The image on is now at a 30 degree angle with the camera map appearing as if it has rotated 30 degrees counter clockwise. The direction of travel in this map is 30 degrees. The version used in this study was identical to that in Coyne et. al. 2024, it had 18 practice items with feedback and then 24 trials without feedback. The practice feedback always showed the correct response direction, and the first 9 trials had some additional animation where a helicopter icon on the reference map rotates in 15 degree increments while simultaneously showing how the camera view would change with that rotation. The version in this paper (unlimited time and computer-generated images) is different from the version that is currently part of the ASTB (per item time limit and satellite images).

### Spatial apperception task/airplane attitude

**Fig. 2:**
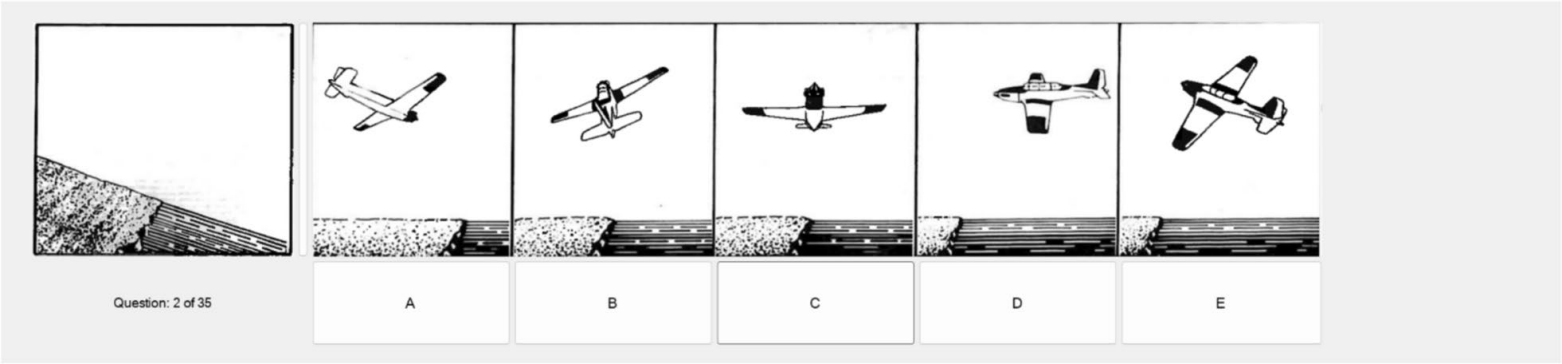
Sample trial from the spatial apperception/airplane attitude task (correct answer is B)

The Spatial Apperception Task (name used by the US military), which we will refer to as the Airplane Attitude Task throughout this paper (Fig. 2) shows the out the window view of an aircraft at a specific attitude flying toward varying terrain features (e.g., water). The objective is to identify which of several aircraft in different attitudes (roll, pitch, and yaw) over terrain would create that out the window perspective. A version of this test was originally used in aviation selection during WWII, and is still currently being used by the US Army for measuring spatial ability in aviation applicants. The version used in this study used items from the previous version of the ASTB (last used in 2013). Participants had 10 min to answer as many trials as they could (up to 35 items).

### Direction orientation task

**Fig. 3:**
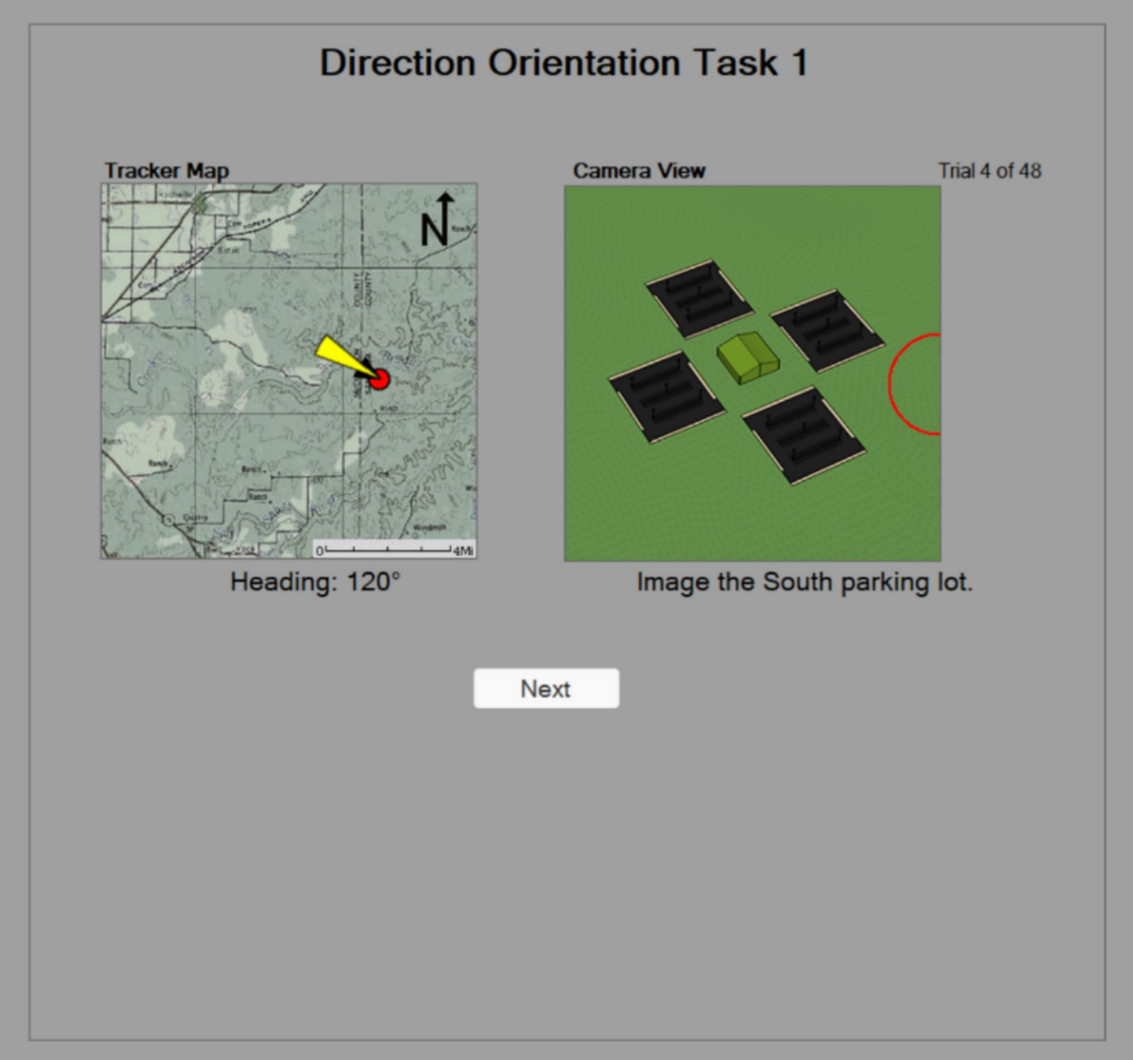
Sample trial from the direction orientation task (correct answer is 2 o’clock position)

The Direction Orientation Task (Fig. 3) is designed to measure orientation, each of the 48 trials show two images, one with a map showing a vehicle’s heading and the other represents the view from the vehicle's forward-facing camera. The objective is to identify one of four parking lots in the camera view. The parking lots are at the cardinal positions. Each trial asks participants to “image” a specific parking lot (e.g., South) by clicking on that lot. If the vehicle is traveling a cardinal direction e.g., West then the West parking lot is at the top of the camera view. The sample trial in Fig. 3. has the vehicle heading 120 degrees in this case there are two parking lots on the top. As mentioned earlier a mathematical solution could be applied to the trial in Fig. 3 in which you compute the difference between the heading and the target lot (180–120 = 60). The target lot is an additional 60 degrees from the heading which corresponds to the correct lot’s 2 O’clock position in the camera view. Participants had 12 practice trials with feedback, followed by 48 trials without feedback. The 48 trails equate to every combination of heading (12) and target parking lot (4). There was unlimited time to complete the test; however, participants were instructed to answer as quickly and accurately as possible.

### Cube comparison and paper folding

Following the three aviation specific spatial ability tasks participants completed two additional general spatial ability tasks. The cube comparison and paper folding tasks were included to have a more diverse set of spatial ability measures (Elkstrom et al., 1976). The paper folding task was completed first. In the paper folding task participants are shown an image in which sheet of paper is folded along different lines, after a series of folds a hole is punched through the paper. Participants were tasked with determining where the hole(s) would appear once the paper is unfolded. Each item had five possible response choices. There were two blocks of ten trials and participants had three minutes to answer as many trials in each block as they could.

The cube comparison task displays two cubes in which three sides are visible. Each side has a unique letter, symbol, or number on it and participants must determine if the two cubes are the same (but in a different orientation) or different. There were two blocks with 21 trials each and a three minute time limit for each block.

### Mental counters

**Fig. 4:**
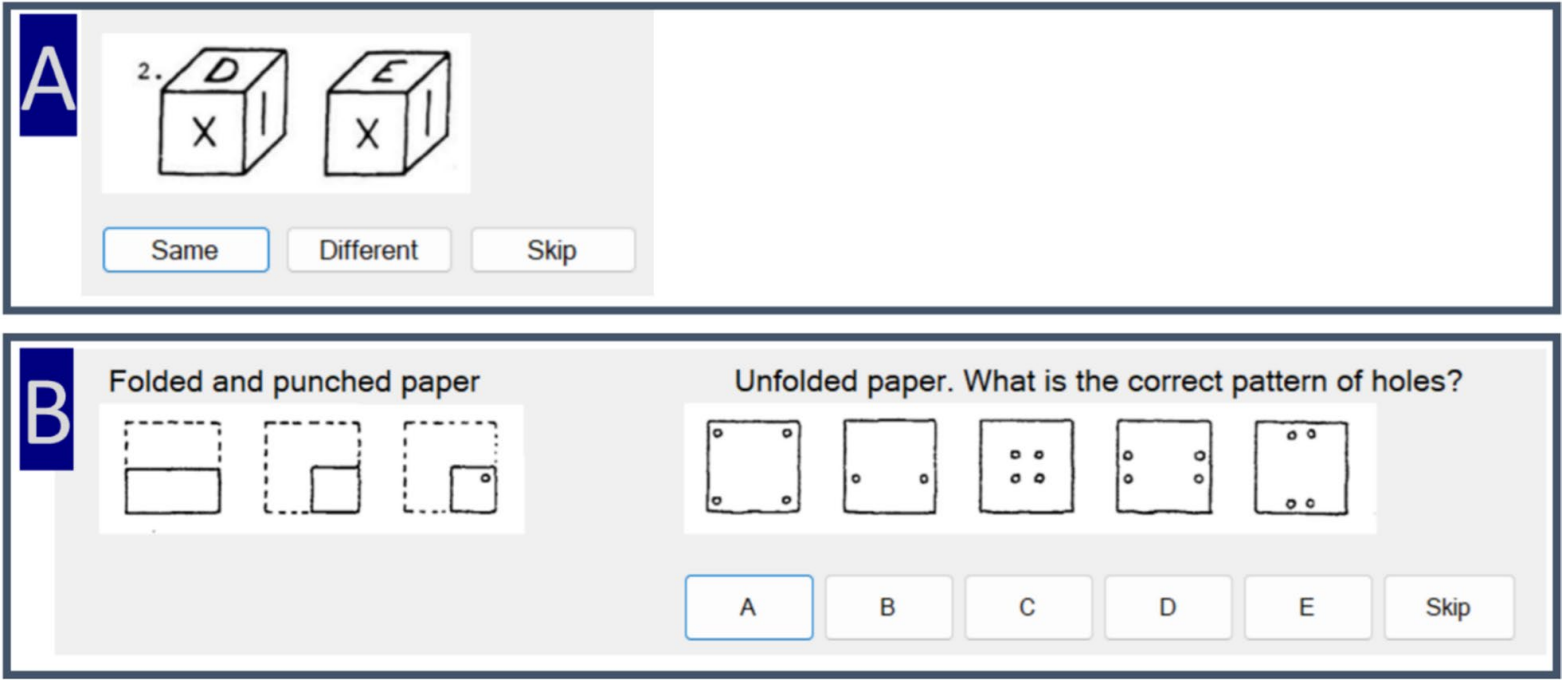
**a** Sample trial from the Cube Comparison task. The two cubes are different. **b** Sample trial from the Paper Folding task. The correct response is D

The Mental Counters task, is a working memory task which is part of the Navy’s Armed Services Vocational Aptitude Battery which is used to select and classify enlisted recruits was also included in the study. Mental counters is an updating task in which individuals maintain 3 numbers in working memory. Updating tasks are ones which a stimulus must be monitored for new information which must then be encoded and replace the old information. Each trial contains a sequence of boxes flashing above or below three lines in the center of the screen. When a box flashes above the line the individual adds one to that number when it flashes below they subtract one from that position. Each position starts with an initial value of five. A trial is scored as correct when all three numbers are correctly reported. There was a total of 32 trials. The speed and length of the sequences varied across trials (all participants saw the same trials); however, the test has become an official test and specifics on timing and duration are considered proprietary (Fig. 4).

### Stroop and flanker double conflict

**Fig. 5:**
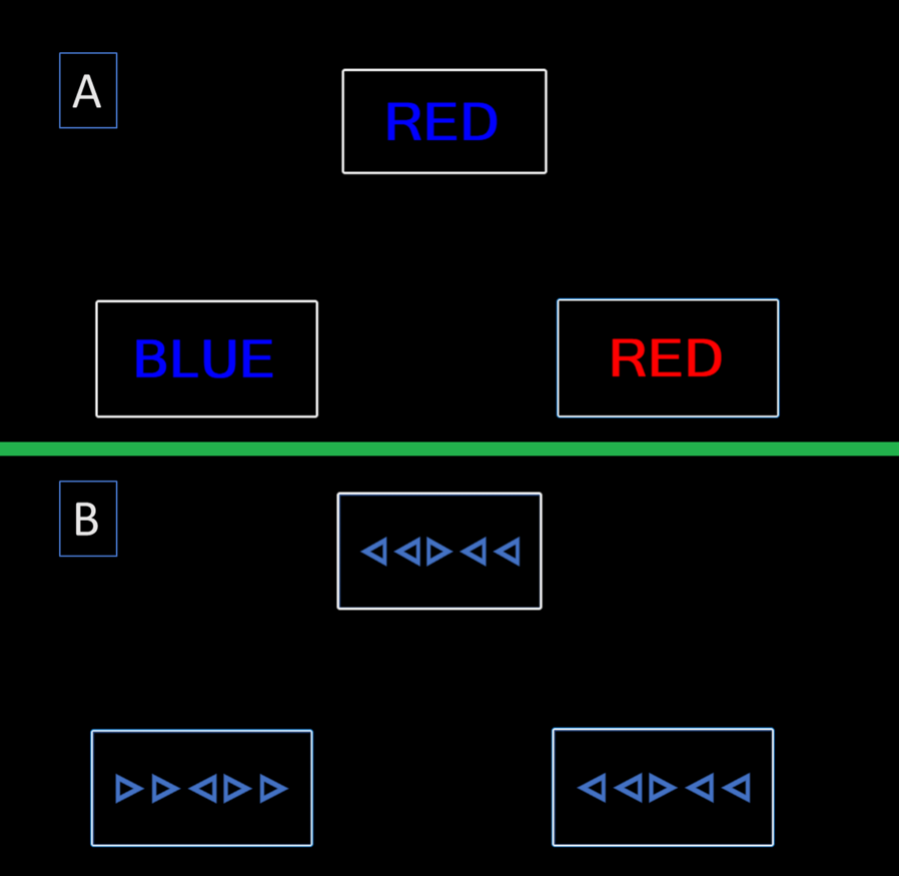
**a** Sample trial from the Stroop Double Conflict Task. Objective is to match the font color on the top to the name of the word below (correct answer is the word blue on the bottom left). **b** Sample trial from the Flanker Double Conflict Task. Objective is to match the direction of the outside arrow on the top with direction of the middle arrow on the bottom (correct answer is the box on the bottom left)

Beyond spatial ability the study also included several other ability measures. These include the Stroop and Flanker double conflict tasks (Burgoyne et al., 2024) which are measures of attention control and had previously demonstrated predictive validity with flight training (Coyne et al., 2024). The Stroop double conflict task (see Fig. 5A) has the participant match the font color of the word on the top with the name of the color below. Both the stimulus and response can either be congruent (font color matches the word) or incongruent. Participants have a 30 s practice to answer as many items correctly as they can. Participants receive a point for each correct response and lose a point for each incorrect response. Following the 30 s practice there is a timed 90 s scored portion. Feedback is provided for every trial in both sessions. The Flanker double conflict task (see Fig. 5B) is similar to the Stroop task in that both the stimulus and response portion can be either congruent (all 5 arrows in the same direction) or incongruent (the outside arrows are in a different direction from the center). The goal of the task is to match the direction of the outside arrow on the top with the direction of the middle arrow in the response. Practice duration, scoring, and feedback are the same as the Stroop task.

### Ravens advanced progressive matrices

Additionally, participants were presented with the odd number items from Raven’s Advanced Progressive Matrices, an established measure of fluid intelligence (Raven & Raven, 2003). Participants had 10 min to solve up to 18 items from the Raven’s test.

## Navy selection and training outcome data

### Aviation selection test battery (ASTB)

Data for tasks listed in the procedure were collected in a lab setting. The lab data was also linked with participants official ASTB (high-stakes) data which they took to qualify for aviation training. Applicants are allowed to take the ASTB up to three times with the most recent attempt being the official score utilized when determining an applicant’s eligibility. All analyses use the final official score. The ASTB subtests scores used in the analysis include the Direction Orientation Task total correct and the Dichotic Listening total correct. No individual subtest of the ASTB is used to make selection decisions. Decisions are based upon three composite scores, which include combinations of the different crystalized and process-based subtests, however only two are relevant to the training being predicted in this paper. The first composite used in this paper is the Academic Qualification Rating (AQR) which we will refer to as the academic composite. The academic composite is designed to maximize prediction of the academic portion of the initial training, known as the Naval Introductory Flight Evaluation. The second composite is the Primary Flight Aptitude Rating (PFAR) or primary composite, is designed to predict training performance during Primary Flight Training, which is where pilots are trained to learn basic flying skills. Both the composites are stanines, a normalized scale that ranges from 1 to 9.

### Naval introductory flight evaluation (NIFE)

All Naval Flight Students complete the Naval Introductory Flight Evaluation (NIFE), which is comprised of four phases. Performance on the ASTB is not meant to predict the first and final phases which involve components such as medical clearances, and water survival. For purposes of this analysis the outcomes of interest are from phase two of the introductory training which is a condensed three-week academic phase in which they must pass exams in five subjects. The introductory training grade is the average score from the 5 exams. The third phase of the introductory training requires students to complete a series of flights in a Cessna 172. Introductory flight score is the normalized grade students receive from their instructors during the flight portion of this initial training.

### Primary flight training

Students who successfully complete the introductory training move on to primary flight training. At this point in training the aviators and flight officers are separated. For purposes of this analysis only data for the Student Naval Aviators are considered, as there are only a small number of Student Flight Officers. Additionally, data from a subset of students were excluded from the analysis as they were part of an experimental flight training program called “Avenger,” which restructured the training, their access to instructors and provided additional access to virtual reality simulators. Students in this program were not assigned a grade for primary otherwise known as the Navy Standard Score (NSS). The standard score is a representation of an individuals’ score during primary flight training relative to the average. The standard score has a center of 50 and is normalized within training groups to allow for an easier comparison across individuals trained at different locations. For Primary Flight training the standard score used in the analysis is based upon flight performance.

## Results

The total sample included 307 officers. In each variable used in the analysis, some missing data was present. The highest rate of missingness was on the Flanker (19%) followed by Folding (14%) and Stroop (12%); all other variables had < 10% missingness. There were some issues with some of the software not loading correctly, particularly with the E-Prime software. There were also some issues linking student’s lab data with their official ASTB data. With respect to the training outcome data, introductory grade and introductory flight are only available for students who complete the academic phase of the introductory training. Primary flight training data used in the analysis only included students who complete the introductory training, were Student Naval Aviators, and completed the traditional version of Primary. In two tasks, Flanker, and Stroop, some participants experienced difficulties in administration and received extremely low scores causing them to be below chance. To identify these individuals and correct their scores to be representative of their true ability level, we used a random forest based multivariate outlier detection method along with predictive mean matching replacement (Chandola et al., 2009; Wright & Ziegler, 2015). This technique was applied to all of the attention measures and three scores on the Dichotic Listening Task were corrected, four scores on the Stroop were corrected, and two scores on the Flanker were corrected. See Table 1 for sample size and descriptive statistics including both the maximum observed and the maximum possible for each scale after applying this correction. The reliabilities are also shown in Table 1. For all of the scales except Stroop and Flanker we report α using the Kuder–Richardson Formula 20 for dichotomous items. For Stroop and Flanker we report estimates using multilevel reliability because, within person, there are varying amounts of repeated measurements in a random order. Two values are reported, ${R}_{\text{KRN}}$ and ${R}_{\text{CN}}$, respectively. The first value (${R}_{\text{KRN}}$) represents the reliability of between-person mean scores on the assessment while the second value (${R}_{CN}$) represents the reliability of within-person item level scores (Shrout & Lane, 2012). For these values, we can follow the general guidelines for interpreting an intraclass correlation to assess these values of reliability. This means that values greater than 0.90 indicate excellent reliability (Koo & Li, 2016).

**Table 1:** Descriptive statistics all scales

Item	N	Mean	Max (obs)	Max (pos)	SD	Skew	Reliability
Airplane attitude	307	13.75	34	35	11.44	− 0.44	0.83
Terrain task	300	15.54	24	24	4.57	− 0.59	0.76
Lab direction	306	40.34	48	48	9.42	− 1.83	0.95
Folding	264	6.83	20	20	6.72	− 0.79	0.78
Cube	306	14.08	42	42	11.56	0.00	0.85
Stroop	269	37.23	63	NA	13.29	− 1.04	0.96, 0.99^1^
Flanker	248	37.66	83	NA	15.81	− 0.23	0.97, 0.99^1^
Counters	307	20.56	32	32	5.81	− 0.64	0.82
Ravens	278	9.76	17	18	3.10	− 0.26	0.72
ASTB direction	302	43.38	48	48	4.70	− 1.93	0.82
ASTB dichotic	302	37.88	44	44	5.15	− 1.46	0.82^2^
Introductory grade	289	93.06	100	100	4.0	− .057	
Introductory Flight Score	283	1.10	1.30	1.35	0.06	1.21	
Primary Score	163	52.08	80	80	11.35	0.19	

^1^Reliability is α for all scales except Stroop and Flanker which are reported using multilevel reliability

^2^Reliability for DLT was estimated using only a portion of the sample (*n* = 169)

Table 2 shows the correlations between all 9 scales using pairwise deletion for missingness; Spearman’s correlation was used to reduce non-normality bias (Bishara & Hittner, 2015). The correlation between the academic composite and Introductory Training Grade was positive, $r(283) = 0.47, p < .001$. We do not provide correlations between the academic composite and any of the subtests as this is proprietary information; however, the academic composite is designed to predict success in ground school and includes the ASTB’s academic subtests (i.e., Math, Verbal, Aviation and Nautical Information) as well as some of the process-based subtests. The correlation between the primary composite and Primary Score was positive $r(160) = 0.26, p < .001$. In Appendix A we have included the correlation between all 9 scales using the same method noted above but instead using the first attempt on the ASTB rather than the final. We include this table for reference; however, since the Navy uses the final attempt on the ASTB for selection purposes we focus on those scores for the purposes of this study.

**Table 2:** Spearman correlations for all variables

	Airplane Attitude	Terrain Task	Lab Direction	Folding	Cube	Stroop	Flanker	Counters	Ravens	ASTB Direction	ASTB Dichotic	Introductory Grade	Flight Score	Primary Score
Airplane Attitude	–													
Terrain Task	0.51***	–												
Lab Direction	0.52***	0.42***	–											
Folding	0.35***	0.35***	0.33***	–										
Cube	0.26***	0.30***	0.27***	0.31***	–									
Stroop	0.13*	0.25***	0.14*	0.32***	0.21***	–								
Flanker	0.26***	0.09	0.31***	0.34***	0.20**	0.33***	–							
Counters	0.21***	0.27***	0.18**	0.38***	0.20***	0.39***	0.33***	–						
Ravens	0.21***	0.30***	0.24***	0.47***	0.22***	0.38***	0.37***	0.41***	–					
ASTB Direction	0.02	− 0.01	0.03	− 0.08	− 0.06	0.04	0.01	− 0.07	− 0.14*	–				
ASTB Dichotic	− 0.05	0	0.07	0.06	0.01	0.15*	0.19**	0.12*	0.13*	0.05	–			
Introductory Grade	0.25***	0.17**	0.26***	0.23***	0.25***	0.22***	0.29***	0.18**	0.22***	− 0.03	0.09	–		
Introductory Flight Score	0.39***	0.24***	0.26***	0.14*	0.11	0	0.20**	0.04	0.06	0.02	− 0.07	0.29***	–	
Primary Score	0.35***	0.15	0.18*	0.15	0.09	0.19*	0.25**	0.17*	0.15	− 0.03	0.03	0.45***	0.49***	–

^*^ p < *.*05; ** p < *.*01; *** < *.*001

To identify factor structure differences among these scales when using high stakes ASTB Direction Orientation Task data as opposed to lab Direction Orientation Task data, we conducted Exploratory Factor Analyses (EFAs) using an oblique rotation to allow for inter-factor correlation estimation. EFAs were conducted on two samples, each sample included the personnel with both high stakes and lab data (*n* = 302), and the lab sample additionally included personnel with only lab data (*n* = 5). This resulted in a high-stakes sample with 302 personnel and a lab sample with complete Navy training data with 306 personnel. Full Information Maximum Likelihood (FIML) was used to account for missing data. We first applied the Hull method (Lorenzo-Seva et al., 2011), the empirical Kaiser criterion (EKC) (Braeken & Van Assen, 2017), and the Comparison Data method (CD) (Ruscio & Roche, 2012) to estimate dimensionality. The results of these three analyses, as well as an evaluation of eigenvalues, were used to determine the number of factors present in each set of data before evaluating the fit of an EFA model.

### Lab exploratory factor analyses

**Fig. 6:**
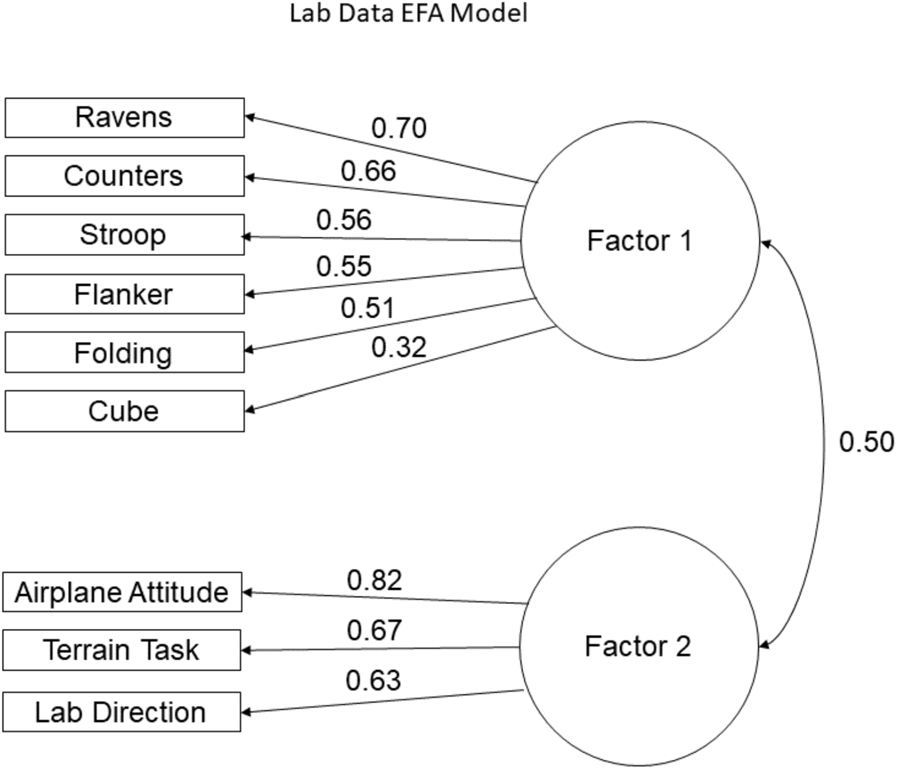
Two factor EFA model using spatial ability measures as well as DOT collected in lab

Using the lab data (*n* = 306), the EKC method indicated a one factor model would fit these data the best, while the Hull and CD methods indicated a two factor model would fit these data the best. The eigenvalues for these data contained two values above 1 (3.45 and 1.30) and all remaining values were below 1. Given these results, we assessed the model fit and structure of a two factor EFA model using an oblique rotation. Figure 6 shows the EFA model using the lab data.

Results indicated that the two factor model fit these data well, ${\chi }^{2}\left(19\right)=31.88,p<.05,\text{RMSEA}=0.05,\text{TLI}=0.96,\text{CFI}=0.98$. In this model, cross loadings were below 0.20 and considerably lower than their respective dominant loadings, indicating that there was no construct contamination (Howard & Henderson, 2023). While there are no recognized standard cutoff values for what constitutes high or low standardized dominant loadings (Peterson, 2000), values above 0.40 are generally considered as high dominant loadings (Comrey & Lee, 2013). Variables with loadings below 0.40, therefore, are generally considered to not be a good representation of the factor. However, provided their cross loadings are not high, they still appropriately load on to their dominant factor.

The first factor was comprised of Ravens, Counters, Flanker, Folding, Cube, and Stroop, where the standardized dominant loadings were all above 0.40, meaning that they load strongly on to this factor, with the exception of the Cube test which had a loading of 0.32. This indicated that the Cube test did not strongly load on to the first factor. The Cube test had a cross loading of 0.18, meaning that it still appropriately loads on to the first factor, but is not a good representation of the first factor. The second factor (correlated to the first at 0.50) was comprised of the Airplane Attitude Task, Terrain Task, and the lab-based Direction Orientation Task, all with dominant loadings above 0.40 and cross loadings below 0.10.

Communalities represent the amount of variance explained in each variable by the factors, where higher communality values are preferable. Similar to factor loadings, there is no standard value which constitutes a good communality value. For the purposes of this study, we considered communality values below 0.30 to be poor fit (MacCallum et al., 1999; Sovey et al., 2022). The two factor EFA model using only lab data had communality values above 0.30 for all variables except for the Cube test which had a communality of 0.19. This indicated that this factor structure did not account well for the variation in the Cube test.

### Lab and high-stakes exploratory factor analyses

**Fig. 7:**
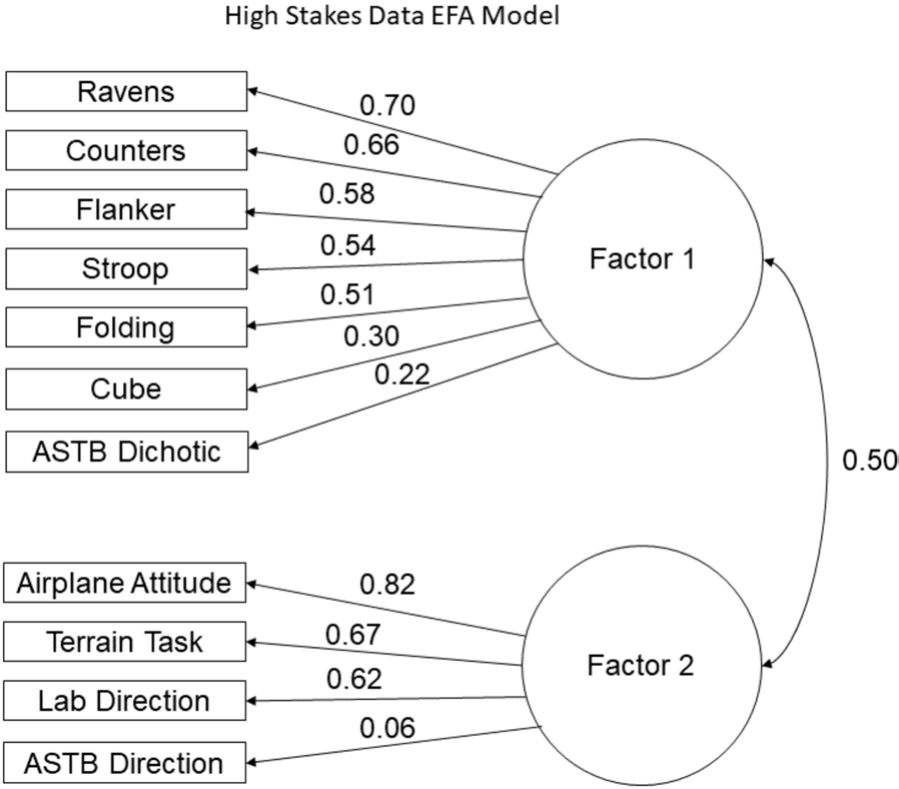
Two factor EFA model using spatial ability tasks and high stakes DOT data

We then analyzed the factor structure using ASTB (high-stakes data) and the lab data. This sample included all officers with high-stakes data (*n* = 302). Using the ASTB and lab data, the EKC method indicated a one factor model would fit these data the best, while the Hull and CD methods indicated a two factor model would fit these data the best. The eigenvalues using the ASTB and lab data contained two values above 1 (3.47 and 1.35), one value very close to 1 (1.11), with all remaining values below 1. Given these results, we assessed the model fit and structure of a two factor EFA model using an oblique rotation. Figure 7 shows the results of this second EFA.

Results showed that the two factor model fit these data well, ${\chi }^{2}\left(34\right)=56.98,p<.01,\text{RMSEA}=0.05,\text{TLI}=0.94,\text{CFI}=0.96$. In this model, all cross loadings were below 0.20 and considerably lower than their respective dominant loadings, with the exception of the high-stakes Direction Orientation. The high-stakes Direction Orientation Task had a low cross loading of − 0.013; however, its dominant loading was also very low (0.06). Even though these values were close, because the dominant loading was so low we do not interpret this as construct contamination. The first factor was comprised of Ravens, Counters, Flanker, Folding, Stroop, Cube, and Dichotic Listening, all with dominant standardized loadings above 0.40, with the exception of the Dichotic Listening Task which had a loading of 0.22, and Cube which had a loading of 0.30. Although the Dichotic Listening Task had a low dominant loading, its cross loading was not comparatively high and was also negative (− 0.11) meaning that the Dichotic Listening Task appropriately loaded on to the first factor and is negatively associated with the second factor. The Cube test had a higher cross loading, 0.19, which, in conjunction with its lower dominant loading, could indicate construct contamination and that it is not representative of the first factor.

The second factor (correlated at 0.50 to the first) was comprised of Airplane Attitude Task, Terrain Task, Lab Direction Orientation Task, and high-stakes Direction Orientation Task. All variables in the second factor, with the exception of the high-stakes Direction Orientation Task, had high dominant loadings (above 0.40) and low cross loadings (< 0.10). The Lab Direction Task had a dominant standardized loading (0.62); however, the high-stakes Direction Task had a very low dominant standardized loading (0.06). This means that the Lab Direction Orientation was a good representation of this factor while the high-stakes Direction Orientation was not.

The communality values for all tests in this model were above 0.30 except for three values. The Cube had a communality of 0.18, the Dichotic Listening had a value of 0.04, and the high-stakes Direction Orientation Task had a value of 0.003. This means that this factor structure did not account well for the variation within these tests. Given these values, we would not consider these variables to be included in the overall factor model. Notably, the communality value for the Lab Direction Orientation Task in this model was 0.44. The factor structure for the variables collected in lab did not change when introducing high-stakes data; however, model fit for high-stakes Direction Orientation Task is poor.

All together, these results present strong evidence that the latent factor assessed using high-stakes Direction Orientation Task responses is not psychometrically equivalent to that of the Direction Orientation Task responses collected in lab. Additionally, the Dichotic Listening Task does not fit well into the overall factor model.

## Discussion

The pattern of results did not match our first hypothesis. The expectation was that Direction Orientation Task in the lab, and the one taken as part of the ASTB would perform similarly; however, the two tests were not even correlated with each other. Direction Orientation Task in the lab loaded onto a spatial factor with the two other aviation selection spatial ability tests (Terrain Task and Airplane Attitude Task). All three of these lab-based spatial ability measures were correlated with training outcomes, whereas the high-stakes Direction Orientation Task was not. While unexpected, these results still provide some support to our hypothesis that the Direction Orientation Task, when taken within the ASTB, may no longer be measuring spatial ability. The authors are aware that some portion of test applicants may be using scrap paper to assist in completing the Direction Orientation Task within the high-stakes environment. Participants in the low stakes testing environment would not have been able to use this strategy.

The authors included the Cube and Folding Tasks as measures of spatial ability and expected them to load on the spatial factor. However, both measures loaded on the general ability factor. The Cube task loaded poorly on the factor; however, the Paper Folding task was a good fit. One possibility is that individuals may have been using a more analytic approach to the Paper Folding task. Indeed, Burte et al. (2019) suggests that non-spatial strategies such as an analytical approach of counting folds and holes are also used when solving items in the task. They suggest that the task is not purely a measure of spatial visualization. This may be why the task loaded on the general factor and demonstrates some of the challenges with process-based measures.

The results for the new attention control measures did match our hypothesis. The new attention control measures were significantly correlated with some of the training outcomes, whereas the attention measure in the high-stakes environment, the Dichotic Listening Task, was not. The ASTB Dichotic Listening Task was correlated with the attention measures and did load on to the same factor in the EFA; however, the structural fit was poor and indicated that some variation in the listening task was not explained by the factor structure.

Some individuals who take the official ASTB are highly familiar and practiced with portions of the test like the Direction Orientation Task and Dichotic Listening Task even if it is their first official test attempt. In preparing for the official test some individuals learn ways to leverage non-spatial solutions to Direction Orientation Task prior to ever having taken the ASTB. In most cases individuals participating in the study had taken the official ASTB over a year prior to the lab-based study. The familiarity that they acquired in preparation for the ASTB may have been lost over that time. The lack of familiarity may explain why Direction Orientation Task in the lab appears to assess spatial ability (and predict training outcomes), whereas Direction Orientation Task scores from the ASTB do not. The results also support the concerns Ackerman (2022) raised about process-based measures.

One explanation for these results is that process-based measures function by having individuals perform either a novel operation or use novel stimuli (Ackerman, 2022). The degree to which practice increases test specific skills, versus an increase in the construct being assessed reflects bias with the measure. While there is a growing body of the literature on effects of re-testing in high-stakes environments (e.g., Lievens et al., 2007; Sibley, 2024), there has not been a sufficient look into the impact of practice which occurs before even the initial test. The sophistication of online resources available to potential aviation applicants as evidenced by their ability to quickly generate unofficial versions of subtests such as Terrain Task within weeks of it appearing suggest that simply looking at re-testing is not sufficient. Examining how repeated exposure and practice can introduce measurement bias and impact the effectiveness of a test should be a concern within high-stakes tests such as the ASTB.

One of the additional goals of the study was to further evaluate the new Terrain Task. The data from this study provide some additional evidence for the effectiveness of the Terrain Task as a potential replacement to Direction Orientation. However, the correlations of the Direction Orientation Task in the lab with the same training outcomes limit the value of these results when making decisions about future versions of the ASTB. As the US Navy considers replacing the Direction Orientation Task as a measure of spatial ability, it should look beyond assessing test–retest reliability for future spatial ability measures. An assumption should be made that some applicants will have had practice with any new test prior to even their initial score on the ASTB. It is likely that practice on the Direction Orientation Task increases test specific skills not relevant to the spatial ability construct, especially given that non-spatial strategies have been shared for the task. While the Terrain Task is a more flexible test given the potential for unlimited number of items, it is unclear how practice on the Terrain Task might change the test’s effectiveness. With respect to the Direction Orientation Task, participants can practice on both the process being measured, as well as with the exact stimuli they will see in the official high-stakes test. The Terrain Task being evaluated within the ASTB has multiple forms, and a large item bank, and while applicants can practice unofficial versions, they do not have access to the actual test items or maps. Thus, when practicing unofficial versions of Terrain Task applicants are being exposed to the process of solving terrain association problems not the test stimuli. Further methods which remove the visual-spatial comparison between the two maps in the Terrain Task have not been identified. These key differences between the tests could allow the Terrain Task to retain its effectiveness even with practice; however, this is an empirical question which has not yet been evaluated.

To a lesser extent this paper also looked at measures of attention, another process-based intelligence measure that is currently assessed within the ASTB. The ASTB relies on the Dichotic Listening Task to measure attention. There are some practical concerns and potential vulnerabilities with this test which potentially hinder its validity. The specifics of these vulnerabilities cannot be shared as the test is still in active use and disseminating that information could impact the Dichotic Listening subtest’s effectiveness. The Dichotic Listening Task was previously included in the Air Force pilot selection battery but has subsequently been dropped.

The study also found additional evidence on the effectiveness of two new attention control measures in predicting flight training outcomes. However, as with all the lab-based measures there was the benefit of the participants in this study being naïve to the task and stimulus. The extent to which these tests depend on a lack of familiarity with the stimuli will ultimately determine whether they are good candidates for use in aviation selection. One disadvantage these double conflict tasks have compared to the Terrain Task is that, if they are replicated by motivated participants, it will allow participants to practice with both the stimulus and the process. The scoring of these tasks, however, does not have a fixed celling as participants are given as many trials as they can answer within the time limit. As with Terrain Task the durability of these tasks under repeated practice needs to be further evaluated.

The concerns raised by this paper extend beyond the ASTB and apply more generally to any high-stakes situations in which processed based tests may be used to measure individual differences. The ASTB’s composite scores which are comprised of both crystalized and process-based measures still predict training outcomes. Test developers routinely evaluate and replace crystalized test items to ensure the test retains its reliability and effectiveness. High-stakes test developers should take a deeper look at all process-based measures. Learnability and familiarity are issues beyond that which can be evaluated solely by investigating a test’s reliability with repeat exposure. Results obtained in lab settings such as those found in this paper must be cautiously considered as performance with a “naïve” sample may not reflect performance once the test is part of an official high-stakes battery. The challenge is that while attention control and spatial ability are important constructs in predicting training outcomes, their effective measurement may hinge on a naivety that can no longer be guaranteed in high-stakes testing. The assumption should be that motivated applicants will replicate any new test that is introduced and be heavily practiced on those tests prior to taking the official battery. This assumption runs counter to the approach test developers have taken in the past. Further research on repeated and deliberate practice in process related measures needs to be done if these measures are going to be used effectively in high-stakes testing environments in the future.

## Data Availability

The official Navy data used in this study are owned by two different Navy Commands, and restrictions apply to the availability of these data. The data from the laboratory tasks are available from the corresponding author upon reasonable request.

## Appendix A

**Table:**

Table: Spearman Correlations for All Variables on First Attempt														
	Airplane attitude	Terrain task	Lab direction	Folding	Cube	Stroop	Flanker	Counters	Ravens	ASTB direction	ASTB dichotic	Introductory Grade	Flight score	Primary score
Airplane Attitude	–													
Terrain Task	0.51***	–												
Lab Direction	0.52***	0.42***	–											
Folding	0.35***	0.35***	0.33***	–										
Cube	0.26***	0.30***	0.27***	0.31***	–									
Stroop	0.13*	0.25***	0.14*	0.32***	0.22***	–								
Flanker	0.26***	0.09	0.31***	0.34***	0.20**	0.35***	–							
Counters	0.21***	0.27***	0.18**	0.38***	0.20***	0.39***	0.33***	–						
Ravens	0.21***	0.30***	0.24***	0.47***	0.22***	0.38***	0.37***	0.41***	–					
ASTB Direction	0.09	0.11	0.12*	0.07	0.04	0.12	0.13	0.07	− 0.04	−				
ASTB Dichotic	0.03	0.05	0.17**	0.06	0.10	0.20**	0.30**	0.17**	0.15*	0.11*	–			
Introductory Grade	0.25***	0.17**	0.26***	0.23***	0.25***	0.23***	0.29***	0.18**	0.22***	0.07	0.14*	–		
Introductory Flight Score	0.39***	0.24***	0.26***	0.14*	0.11	0.00	0.20**	0.04	0.06	0.09	− 0.06	0.29***	–	
Primary Score	0.35***	0.15	0.18*	0.15	0.09	0.19*	0.24**	0.17*	0.15	− 0.03	0.09	0.45***	0.49***	–

**p* < .05; ***p* < .01; *** < .001

## Abbreviations

AQR: Academic qualification rating
ASTB: Aviation selection test battery
EFA: Exploratory factor analysis
NIFE: Naval flight introductory evaluation
NFS: Naval flight students
NSS: Navy Standard Score
PFAR: Pilot flight aptitude rating
